# Empagliflozin Alleviates Left Ventricle Hypertrophy in High-Fat-Fed Mice by Modulating Renin Angiotensin Pathway

**DOI:** 10.1155/2022/8861911

**Published:** 2022-01-18

**Authors:** Juliana Cordovil Cotrin, Gabriel Santos Martins de Souza, Tamiris Ingrid Petito-da-Silva, Luiz Eduardo Macedo Cardoso, Vanessa Souza-Mello, Sandra Barbosa-da-Silva

**Affiliations:** Laboratory of Morphometry, Metabolism and Cardiovascular Disease, Institute of Biology, State University of Rio de Janeiro, RJ, Brazil

## Abstract

*Aims*. The cardiobenefits of empagliflozin are multidimensional, and some mechanisms are still unclear. The aim of the present study was to evaluate the effect of treatment with empagliflozin on biometric parameters and gene expression in the local cardiac RAS, oxidative stress, and endoplasmic reticulum pathways in a mouse model. *Main Methods*. Forty male C57BL/6 mice were fed with control (C) or high-fat (HF) diets for 10 weeks. After that, the groups were redistributed according to the treatment with empagliflozin—CE or HFE. The empagliflozin was administered via food for 5 weeks (10 mg/kg/day). We performed biochemical analyses, blood pressure monitoring, oral glucose tolerance test, left ventricle (LV) stereology, RT-qPCR for genes related to classical and counterregulatory local RAS, oxidative stress, and endoplasmic reticulum stress. *Key Findings*. In comparison to HF, HFE decreased body mass and improved glucose intolerance and insulin resistance. The cardiac parameters were enhanced after treatment as expressed by decrease in plasma cholesterol, plasma uric acid, and systolic blood pressure. In addition, LV analysis showed that empagliflozin reduces cardiomyocyte area and LV thickness. The local RAS had less activity of the classical pathway and positive effects on the counterregulatory pathway. Empagliflozin treatment also decreased oxidative stress and endoplasmic reticulum stress-related genes. *Significance*. Our results suggests that empagliflozin modulates the local RAS pathway towards alleviation of oxidative stress and ER stress in the LV, which may be a route to its effects on improved cardiac remodeling.

## 1. Introduction

Cardiovascular diseases (CVDs) are the leading cause of death globally, and obesity is the third cause of death from noncommunicable disease. The latter is an established risk factor for diabetes mellitus type 2 (DM2) and leads to development of CVDs, affecting the cardiac structure and function [1].

The current treatments for DM2 are based on strict glycemic control, without substantial effect on CVD reduction, often leaving a residual vascular risk [2]. In 2008, the Food and Drug Administration issued guidelines to ensure that DM2 drugs had cardiovascular safety as a prerequisite. In this context, type 2 sodium and glucose cotransporter inhibitors (SGLT-2i) were developed as a new class of antihyperglycemic agents that inhibit, concomitantly, the reabsorption of glucose and sodium in the renal proximal convoluted tubule [3]. The ensuing glycosuria and natriuresis translate into an approximate 0.5 to 1.2% reduction in glycated hemoglobin, a 4-5/2 mmHg reduction in blood pressure (BP), and a 2 to 3 kg reduction in body mass [4].

The cardiobenefits of SGLT2i are multidimensional, and empagliflozin was the first drug in the SGLT2i class to show a significant reduction in CVD risk [5], presumably related to the weight loss [6]. Empagliflozin has also been reported to decrease cardiac fibrosis, arteriolar wall, and cardiovascular oxidative stress levels in obese mice, in addition to reduced left ventricular (LV) weight [7] and diameter of cardiomyocytes [8].

Another target of empagliflozin is the renin-angiotensin system (RAS). About 14% of patients with DM2 and resistant hypertension have a partial or complete autonomous aldosterone hypersecretion of RAS [9]. In the EMPA-REG-OUTCOME trial, such activation cannot be easily demonstrated since the vast majority of patients received treatments that block RAS activity. Therefore, the reduction in CV mortality could be a consequence of pharmacological interactions (SGLT2i+RASi) [10].

RAS has also been described as capable of enhancing the production of intracellular reactive oxygen species (ROS) by stimulating Nox2 and Nox4 and inhibiting SOD and catalase [11]. This stress increases cardiac hypertrophy as well as blood pressure (BP), both factors increasing the stimulus for endoplasmic reticulum (ER) stress [12]. However, it is known that there is an increase in both classical and nonclassical RAS pathway activities after SGLT2i use [13]. Besides, the local RAS activity, in cardiac tissue, activates the nonclassical pathway via Ang(1-7), leading to vasodilation, anti-inflammatory effects, and positive inotropic effects [14].

Although it was previously described that SGLT2i improves cardiac function and reduces cardiac fibrosis, there is a lack of studies evaluating the role of SGLT2i on cardiac RAS. The hypothesis that the cardioprotective effect of SGLT-2i is mediated by interaction with RAS is intriguing, and the present study was aimed at investigating whether empagliflozin treatment affects local RAS so that it would contribute to the reduction of cardiac changes caused by high-fat diet and the consequent changes in body mass, blood glucose, and BP, in addition to a reduction of oxidative stress and ER stress.

## 2. Material and Methods

### 2.1. Animals and Diets

The study was approved by the local ethics committee CEUA/031/2017, and the protocol followed the recommendations of the Guide for the Care and Use of Laboratory Animals of the National Institutes of Health (NIH Publication number 85-23, revised in 1996). We used three-month-old male mice (C57BL/6) maintained in ventilated cages under controlled conditions (NexGen System, Allentown Inc., PA, USA, 20 ± 2°C, 60%u, and 12 h/12 h dark/light cycle), with free access to food and water.

**Figure 1:**
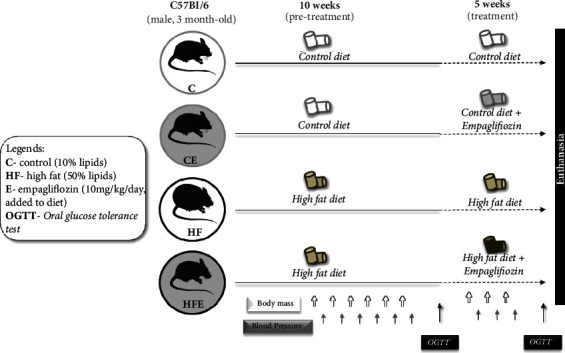
Experimental design. C: control; CE: control with empagliflozin; HF: high fat; HFE: high fat with empagliflozin.

Forty male mice were randomly divided into two groups (*n* = 20/group): control diet (C, 10% energy from lipids) or high-fat diet (HF, 50% energy from lipids), for ten weeks. The diets were provided by PragSoluçoes (Jau, SP, Brazil) according to the recommendations of the American Institute of Nutrition for rodents (AIN93 M) [15] (Table 1). After ten weeks, the animals were divided into two new groups to receive treatment with empagliflozin (Jardiance, Boehringer Ingelheim Pharmaceuticals,10 mg/kg/day mixed with the diet) for an additional five weeks. The groups were formed (*n* = 10/group) as follows: C group (control diet), CE group (control diet plus empagliflozin), HF group (high-fat diet), and HFE group (high-fat diet plus empagliflozin) (Figure 1).

### 2.2. Biometric Analysis

Body mass was measured once a week on a precision balance (model B320H, Shimadzu, Brazil). Food and water intake were measured daily and recorded as the difference between the supplied food/water and the amount left in the cage after 24 h.

### 2.3. Oral Tolerance Test (OGTT)

Oral glucose tolerance test (OGTT) was performed before and after the treatment. After a 6 h fast, 1 g/kg glucose was administered by orogastric gavage (25% in sterile saline, 0.9% NaCl). Blood glucose measurement from the tail tip using a glucometer (Accu-Chek Go, Roche Diagnostic, Mannheim, Germany) was taken before glucose administration (time 0) and thereafter at 15, 30, 60, and 120 min.

### 2.4. Plasma Analyses

The fasting insulin concentration was measured by ELISA (enzyme-linked immunosorbent assay) using a commercial kit (Cat. # EZRMI13K, Millipore, Missouri, USA) and with TP Reader Thermoplate equipment (Tek Instruments, Inc., Highland Park, USA). Plasma uric acid and total cholesterol were quantified by an enzymatic colorimetric method using 25 *μ*L of plasma in an automated spectrophotometer and commercial kits (Bioclin System II, Quibasa Ltda., Belo Horizonte, MG, Brazil).

### 2.5. HOMA-IR (Homeostatic Model Assessment of Insulin Resistance)

The insulin resistance which was analyzed by HOMA-IR was used: HOMA − IR = I0 × G0/22.5, in which I0 is the fasting insulin (*μ*u/mL) and G0 is the fasting blood glucose (mg/dL).

### 2.6. Urinary Analyses

The urine was collected for 24 h in metabolic cages for individualized animals and stored at -20°C until the time of analysis. An aliquot of 25 *μ*L was diluted in water at 1 : 10, and the uric acid was determined with an automated spectrophotometer and commercial kits (Bioclin System II, Quibasa Ltda., Belo Horizonte, MG, Brazil).

### 2.7. Systolic Blood Pressure (SBP)

SBP was measured by the noninvasive caudal plethysmography method using a calibrated tail-cuff system (Insight, Ribeirao Preto, SP, Brazil). Mice were kept warm using a warming pad and were acclimatized and trained at least two weeks (excluded from the analysis) before the actual measurements to be used for data analysis were taken. A mean of 3 measurements per day/animal was used to express the final systolic BP.

### 2.8. Euthanasia

Mice were fasted for 6 h and anesthetized intraperitoneally with ketamine (240 mg/kg) and xylazine (30 mg/kg), after which the blood was collected via a cardiac puncture and centrifuged at room temperature (712 g for 15 min). The plasma obtained was then stored individually at -80°C for the under-described analysis.

### 2.9. Tissue Extraction and Analyses

Scherle's method [16] was applied to weigh the heart and the LV, dissected through the identification of the valvar plane. The samples were frozen and stored at -80°C for future molecular analyses or fixed in formaldehyde. Half of the fixed samples were used for collagen analyses, while the other half was embedded in Paraplast Plus (Sigma-Aldrich, St. Louis, USA), sectioned at 5 *μ*m thickness, and stained with hematoxylin and eosin for microscopy. The observations and digital photomicrographs were obtained with a Nikon microscope (model 80i) and DS-Ri1 digital camera (Nikon Instruments, Inc., New York, USA).

### 2.10. Stereology and Morphometry

Stereological analysis was performed to estimate the mean area of cardiomyocytes (A[car]), and LV thickness was assessed by morphometric analysis.

A 36-point test system produced with STEPanizer (http://www.stepanizer.com) was used for data acquisition. Thus, the volume density (Vv) of the cardiomyocytes was estimated by the ratio between the partial points that touched them (Pp) and the total points of the test area (Pt), following the formula Vv = Pp ÷ Pt. The mean cross-sectional area (*A*) was estimated as *A* = VV ÷ 2Qa. Qa corresponds to the total number of cardiomyocyte nuclei in the test area Qa = *N* ÷ At (where *N* is the number of cardiomyocytes and At is the test area). LV wall thickness was measured on the compact portion of the wall at the atrioventricular valve plane, using ImagePro software and LV thickness of 5 animals in each group was analyzed.

### 2.11. Concentration of Total Collagen

The LV obtained was finely minced, washed three times for 30 minutes in distilled water, immersed in acetone for 24 hours, and submitted to two changes of 24 hours each in 40 mL of chloroform : methanol (2 : 1, *v*/*v*) at room temperature. The solvent was decanted, and after incubation at 60°C for 30 minutes, a preparation of dry and defatted myocardial tissue was obtained. Next, about 4-8 mg of this preparation was hydrolyzed in 1 mL 6 N HCl for 18 hours at 118°C, and the concentration of total collagen was then determined by a colorimetric hydroxyproline assay, using trans-4-hydroxy-L-proline (Sigma-Aldrich, H54409) as the standard. Results were expressed as micrograms of hydroxyproline per milligram of dry, defatted tissue.

### 2.12. Quantitative Real-Time PCR (RT-qPCR)

RT-qPCR was performed to detect mRNA expression of genes related to oxidative stress, ER stress, and RAS. Total RNA of LV was extracted using TRIzol reagent (Invitrogen, CA, USA). mRNA concentration was determined by spectroscopy using NanoVue (GE Life Sciences). Then, 1 *μ*g of RNA was treated with DNAse (Invitrogen). The cDNA was synthesized using oligo (dT) primers and reverse-transcriptase Superscript III (Invitrogen, CA, EUA). The Biorad CFX96 cycler and the SYBR Green mix (Invitrogen, CA, USA) were used. The endogenous *β*-actin was used to standardize the expression of the selected genes. PCR reactions were performed following a polymerase denaturation and activation program (4 min at 95° C), with 44 cycles, each consisting of 95°C for 10 s and 60°C for 15 s, followed by a fusion curve (60-95°C, with a heating rate of 0.1°C/s). Negative controls consisted of wells in which the cDNA was substituted for deionized water. The signals were quantified using the *ΔΔ*Ct method to estimate the difference between the number of target gene cycles and the endogenous control. Sense and antisense primer sequences used for amplification are described in Table 2.

### 2.13. Data Analyses

To analyze both groups in the pretreatment phase, the Student *T*-test was used. For analysis of the four groups during treatment, the difference was analyzed with Brown-Forsythe and Welch one-way ANOVA and Tamhane T2 posttest (GraphPad Prism, version 8, CA, USA). We tested the possible interactions between diet and treatment with a two-way ANOVA. Results were presented as mean ± standard deviation, and a *p* value < 0.05 was considered significant.

## 3. Results

### 3.1. Body Mass Gain and Fasting Glucose

During pretreatment, the BM was higher in the HF than in the C group (+14%, *p* < 0.001); also, HF compared to C group showed a -16% decrease in food intake (*p* < 0.0001). After empagliflozin, BM decreased 7% in HFE when compared with its counterpart (*p* = 0.005), whereas, in food intake, HFE showed a 12% increase in comparison with HF (*p* < 0.0001). Two-way ANOVA revealed that the diet influenced the final BM, accounting for 58.86% of its total variance (*p* < 0.0001). The treatment and the interaction between diet and treatment also influenced this parameter, albeit with low intensity (*p* = 0.01 and *p* = 0.007, respectively).

In fasting glucose, a difference was observed between C and HF groups, with an increase of 78% (*p* = 0.0002). In the treated groups, differences were observed in HFE (-23% *p* = 0.016) compared to its untreated counterpart. The factor that had the most influence on this result was the diet (two-way ANOVA, 70.74%, *p* < 0.0001) (Table 3).

### 3.2. Glucose Tolerance and Insulin Resistance

Regarding glucose tolerance, a higher area under the curve (AUC) was observed in the HF than the C group before treatment (+25%, *p* = 0.02). However, after treatment, AUC in the HFE decreased significantly in comparison with their counterpart (-5%, *p* = 0.04).

The plasma insulin increased in the HF when compared with the C group (108%; *p* = 0.007). On the other hand, the HFE presented an improvement in this parameter in comparison with the HF group (-56%, *p* = 0.01).

The HOMA-IR index showed that the HF group had significantly higher insulin resistance than the C group (+165%, *p* = 0.0079). Treatment with empagliflozin improved insulin resistance in HFE compared to HF (-46%, *p* = 0.0486) (Table 3).

Two-way ANOVA revealed that diet accounted for 77.06% OGTT and for 51.65% in HOMA-IR index (*p* < 0.0001).

### 3.3. Urinary Volume and Glucose Urinary

Empagliflozin increased urinary volume in both treated groups compared to their untreated counterparts (+88%, *p* = 0.003 in HFE and 42%, *p* = 0.02 in CE).

There was no difference in glucose urinary between the C and HF groups (*p* > 0.99). On the other hand, in the treated groups, a significant difference was observed both in the CE group and in the HFE group compared to their untreated counterparts (*p* < 0.01). Two-way ANOVA showed that diet (*p* < 0.001) and treatment (*p* < 0.001) separately influenced urinary volume, as shown in Table 3.

### 3.4. Plasma Cholesterol

Total cholesterol was 13% higher in the HF when compared with the C group (*p* = 0.008). In contrast, the plasma cholesterol of the HFE presented a significant reduction when compared to the HF group (-19%, *p* < 0.001, Table 3).

Two-way ANOVA showed that the plasma cholesterol was influenced simultaneously by diet and empagliflozin (54.9% *p* < 0.0001).

### 3.5. Uric Acid Analyses

Uric acid plasma of the HF presented significantly higher concentration than the C group (+24%, *p* = 0.012); however, the HFE showed reduction comparison with the HF group (-17%, *p* = 0.017). An opposite result was observed in the CE in which plasma uric acid increased by 7% (*p* = 0.027), compared to its counterpart.

In urinary, uric acid decreased -42% in the HF compared to the C group (*p* = 0.045), the HFE increasing its concentration (+53%, *p* = 0.046) compared with the HF group (Table 3).

Two-way ANOVA showed that diet, empagliflozin, and the interaction between them influenced plasma uric acid, but it is noteworthy that the interaction accounted for 46.04% making it the most influential factor (*p* < 0.001). Diet was the factor that most influenced the urinary uric acid data (53.77%, *p* < 0.001).

### 3.6. Systolic Blood Pressure (SBP)

**Figure 2:**
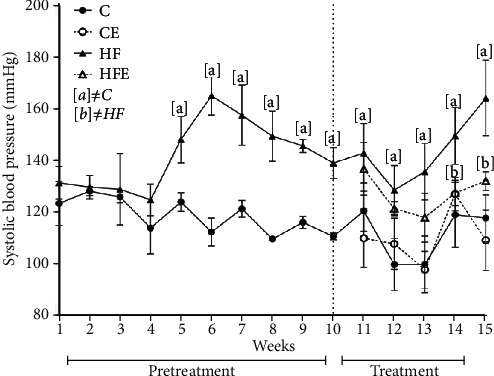
Systolic blood pressure (mmHg) pre- and during treatment. Data presented with mean ± standard deviation, *n* = 5. C: control; CE: control with empagliflozin; HF: high fat; HFE: high fat with empagliflozin. [a] ≠C; [b] ≠HF. *p* < 0.05; one-way ANOVA and posttest Tamhane T2.

Before treatment, significant difference was observed in SBP between the HF and C groups (+25%, *p* < 0.001), and this difference did not change until the end of the experiment (+39%, *p* = 0.004). Moreover, after treatment, the HFE showed lower SBP levels than the HF (-20% *p* = 0.04). There was no difference between C and CE groups (*p* = 0.79). Diet, treatment, and the interaction between them influenced BP, but diet had the most pronounced effect (two-way ANOVA, 57.13, *p* < 0.001, Figure 2).

### 3.7. Left Ventricle Results

#### 3.7.1. Hydroxyproline Measurement

**Figure 3:**
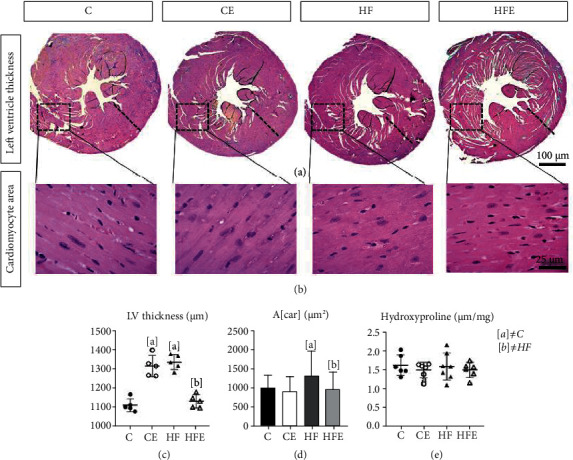
(a) Left ventricular thickness, cross-section caudal view at the valvar plane showing the differences in the wall thickness. (b) Photomicrographs of the cardiomyocytes, paraffin sections stained by hematoxylin and eosin (×600). (c) Left ventricular thickness (*μ*m). (d) Cross-sectional area of cardiomyocyte (A[car] (*μ*m^2^)). (e) Hydroxyproline concentration in dry tissue of left ventricle. All data are presented as mean ± standard deviation, *n* = 5. C: control; CE: control with empagliflozin; HF: high fat; HFE: high fat with empagliflozin. [a] ≠C; [b] ≠HF. *p* < 0.05; one-way ANOVA and posttest Tamhane T2.

The content of total collagen in the myocardium, as determined by a hydroxyproline assay, did not differ significantly between C and HF groups (*p* = 0.99) and HF and HFE groups (*p* = 0.87), as well as between C and CE (*p* = 0.94) (Figure 3).

#### 3.7.2. Left Ventricle Remodeling

Concerning the LV mass, the HF presented a higher LV/tibia ratio than the C group (+17%; *p* < 0.001). On the other hand, the HFE group had a lower LV/tibia ratio when compared with its counterpart (-12%; *p* = 0.017), while the CE LV/tibia ratio compared to the C group did not differ significantly (=0.002, Table 3).

The cardiomyocyte cross-sectional area (A[car]), in the HF, showed hypertrophied cardiomyocytes, with an increase of 30% (*p* = 0.03) compared to that in the C group. On the other hand, A[car] was lower in the HFE when compared to the HF group (-27% *p* = 0.016, Figure 3).

Influenced by these results, the LV thickness increased by 20% in the HF compared to the C group (*p* < 0.001), and empagliflozin treatment decreased LV thickness by 15% (*p* = 0.0001) in the HFE compared to the HF group. Conversely, in the CE, there was an increase of 18% in LV thickness compared to the C group (*p* = 0.001, Figure 3).

Two-way ANOVA showed that diet was the most influential factor in the LV/tibia ratio (50.17%, *p* < 0.001), while the LV thickness was most influenced by diet-treatment interaction (87.52%, *p* < 0.0001), and the A[car] was most influenced by treatment, as a single factor (5.15%, *p* = 0.001).

### 3.8. Gene Expression

#### 3.8.1. Local RAS System

**Figure 4:**
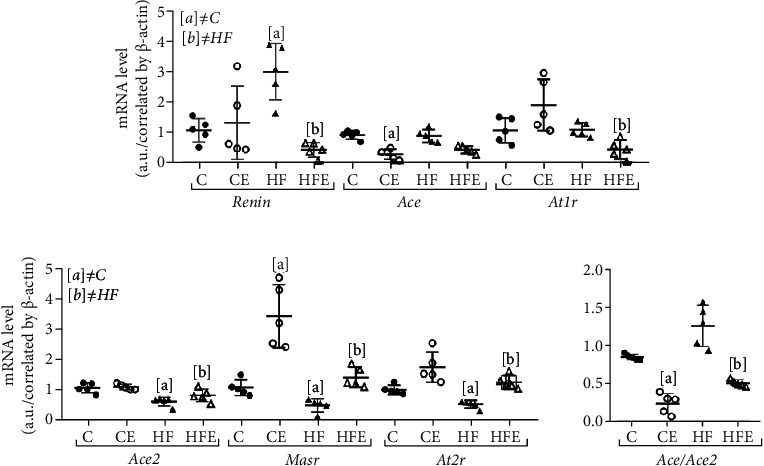
Effect of empagliflozin on mRNA levels of genes involving in renin angiotensin system (RAS). (a) Classic pathway: renin, angiotensin-converting enzyme (Ace), angiotensin II type 1 receptor (AT1r). (b) Contraregulatory pathway: angiotensin-converting enzyme 2 (Ace2), Mas-receptor (Masr), angiotensin II type 2 receptor (At2r). (c) Ace/Ace2 ratio. Data are presented as mean ± standard deviation, *n* = 5. C: control; CE: control with empagliflozin; HF: high fat; HFE: high fat with empagliflozin. [a] ≠C; [b] ≠HF. *p* < 0.05; one-way ANOVA and posttest Tamhane T2.

There was an improvement in local LV RAS with decreased expression of these pathway-related genes after empagliflozin treatment, while its counterregulatory pathway was increased (Figure 4).

Renin is the rate-limiting enzyme of the renin-angiotensin system, and its gene expression was higher in the HF compared to the C group (+179%, *p* = 0.03), whereas the HFE had a decreased renin compared to its counterpart (-86%, *p* = 0.01). ACE is an enzyme that cleaves Ang I, but that was no difference of relative mRNA expression of ACE between the C and HF groups (*p* > 0.99). However, ACE was influenced by empagliflozin treatment, as shown by the decreased expression in the HFE compared to HF group (-52%, *p* = 0.03) and also in the CE compared to C group (-69%, *p* = 0.001).

AT1r is the major receptor for Ang II-mediated cardiovascular functions in mice, and its expression did not differ between the C and HF groups (*p* > 0.99). However, AT1r expression was lower in the HFE than in the HF group (-60%, *p* = 0.03).

Concerning the effects of the counterregulatory axis, AT2r has been recognized as an integrative part of the protective arm of the RAS, and the current results showed 48% reduction in the gene expression of AT2r in the HF group (*p* = 0.004) compared to the C group. On the other hand, its expression in HFE was increased by 136% compared to the HF group (*p* = 0.004). ACE2 is a novel ACE homolog that binds to the MASr, an important receptor of the counterregulating axis. The gene expression of ACE2 was 42% lower in the HF group than in the C group (*p* = 0.01), and after empagliflozin treatment, there was no difference between HF and HFE (*p* = 0.49). MASr decreased by 54% in the HF compared to the C group (*p* = 0.029). Both treated groups had an increase in its expression, +185% in the HFE (*p* = 0.008) and +217% in the CE group (*p* = 0.033) compared to their untreated counterparts.

In the two-way ANOVA, treatment significantly influenced the AT2r, ACE1, and MASr gene levels (*p* < 0.001), while in Renin and AT1r expression, the most expressive stimulus came from an interaction between diet and empagliflozin (*p* = 0.001 and *p* = 0.005, respectively), and ACE2 was influenced only by diet (*p* < 0.0001).

### 3.9. Oxidative Stress System

Oxidative stress is associated with metabolic disorders, such as hypertension, and is conducive to LV hypertrophy and heart failure. However, there are antioxidant enzymes that protect biological systems. Therefore, some antioxidant enzymes were analyzed, and the gene expression of catalase decreased in the CE in comparison with the C (-64%, *p* = 0.003). On the other hand, it was significantly augmented in HFE (+118%, *p* < 0.001) compared with the HF group. Similar results were found in Sod1 (-76%, *p* = 0.002) and Sod2 enzymes (-58%, *p* = 0.015), as both showed significant reductions in the HF when compared with the C group, but in HFE, these enzymes were significantly increased (Sod1: +159%, *p* = 0.02; Sod2: +159%, *p* = 0.029) in comparison with the HF group. Catalase and Sod2 were also increased in the C and CE groups (catalase: +104%, *p* = 0.04; Sod2: +37%, *p* = 0.028).

A role of Nox4 in cardiac and vascular protection has been reported, and it presented higher expression in the HF compared to the C group (+95%, *p* = 0.04), but after treatment, Nox4 expression was significantly reduced in the HFE in comparison with the HF group (-79%, *p* = 0.003).

**Figure 5:**
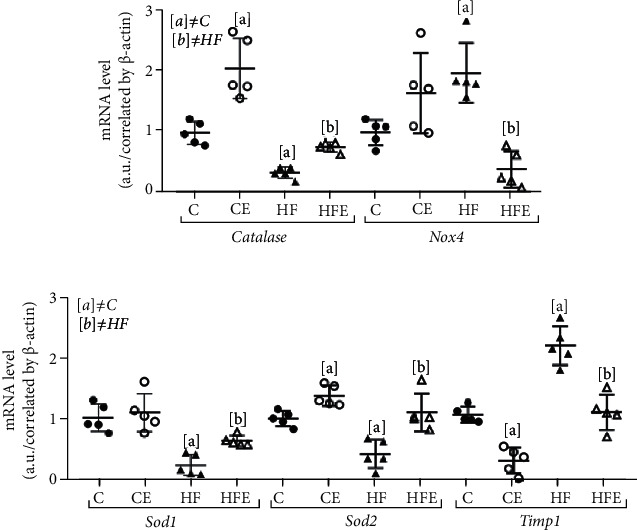
Effect of empagliflozin on mRNA levels of genes involving in oxidative stress. Catalase, NADPH oxidase 4 (Nox4), superoxide dismutase [Cu-Zn] (Sod1), superoxide dismutase [Mn] (Sod2), metallopeptidase inhibitor type 1 (Timp1). Data are presented as mean ± standard deviation, *n* = 5. C: control; CE: control with empagliflozin; HF: high fat; HFE: high fat with empagliflozin. [a] ≠C; [b] ≠HF. *p* < 0.05; one-way ANOVA and posttest Tamhane T2.

Timp1 had higher expression in the HF compared to the C group (+106%, *p* = 0.004) and decreased after treatment (C vs CE: -70%, *p* = 0.002, HF vs HFE: -50%, *p* = 0.003) (Figure 5).

Two-way ANOVA highlighted that the primary effect of Nox4 expression was from an interaction between diet and treatment (58, 35%, *p* < 0.001), while Sod1, catalase, and Timp1 expression diets accounted for 63.05%, 51.38%, and 46.37%, respectively, of their variances (*p* < 0.001), and Sod2 had treatment as its main influencing factor (43.67%, *p* < 0.001).

### 3.10. ER Stress System

Experimental evidence suggests that ER stress deregulation is implicated in heart diseases [17]. Atf4 is a master regulator for ER stress, and we found that its gene expression was increased in the HF compared to the C group (+60%, *p* = 0.004). In contrast, in the treated groups, HFE and CE showed reduced Atf4 gene expression (-56%, *p* = 0.005 and -46%, *p* = 0.019, respectively) compared to their counterparts.

Chop is a widely investigated biomarker involved in ER stress-associated apoptotic signaling in CVD, whose gene expression was enhanced in HF in comparison with the C group (+37%, *p* = 0.01). In turn, empagliflozin treatment decreased its expression in both HFE (-51%, *p* = 0.002) and CE groups (-41%, *p* = 0.001) in comparison with their counterparts.

**Figure 6:**
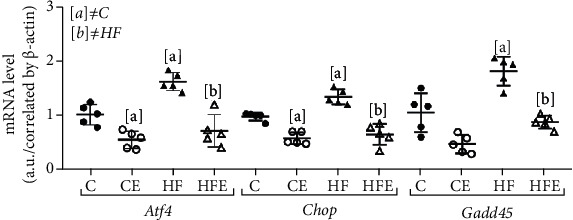
Effect of empagliflozin on mRNA levels of genes involving in endoplasmic reticulum (ER) stress. Atf4: activating transcription factor 4; Chop: Caat-enhancer-binding protein homologous protein; Gadd45: growth arrest and DNA damage-inducible gene 45. Data presented with mean ± standard deviation, *n* = 5. C: control; CE: control with empagliflozin; HF: high fat; HFE: high fat with empagliflozin. [a] ≠C; [b] ≠HF. *p* < 0.05; one-way ANOVA and posttest Tamhane T2.

Gadd45 is a stress signal gene that is expressed in response to physiological or environmental factors. Its expression was augmented in the HF compared to the C group (+73%, *p* = 0.034), but it was lower in the HFE than in the HF group (-52%, *p* = 0.003, Figure 6). Two-way ANOVA revealed that Atf4, Chop, and Gadd45 were influenced by empagliflozin treatment (*p* < 0.001).

## 4. Discussion

This study investigated an experimental mouse model with a high-fat diet that triggered detrimental alterations in SBP, LV thickness, and important metabolic pathways, like local RAS in the LV, ERE, and oxidative stress. On the other hand, the SGLT2 inhibitor, empagliflozin, showed positive results by mitigating most of these alterations.

Empagliflozin treatment reduced BM by 7%, and this can be explained because empagliflozin leads to a continuous excretion of about 60-100 g glucose in the urine, corresponding to a calorie loss which contributes to weight loss [18]. Both glucose and insulin of the treated animals improved, characterizing a decrease in insulin resistance. Insulin resistance could be one factor for damage to cardiac tissue by inducing *de novo* lipogenesis and increased lipotoxicity in target tissues such as the heart [19]. This study showed an influence of empagliflozin on the decrease in total cholesterol; these effects probably stemmed from reduced LDL clearance and reduced intestinal cholesterol absorption [7, 20].

It is given that hyperuricemia is a contributing factor in the development of hypertension, a risk factor for CVD [21]. Our results demonstrated that empagliflozin decreased plasma uric acid but increased its urine excretion. Reductions in uric acid have also been reported in the EMPA-REG OUTCOME trial, and this effect by empagliflozin is possibly due to the increasing renal urate elimination. Almost all of the filtered uric acid is reabsorbed via the urate transporter and the facilitative glucose transporter 9 in the basolateral membrane of the proximal tubule and the consequent increase in uricosuria [22].

More than 50% of hypertensive patients have additional cardiovascular risk factor as diabetes, lipid disorders, overweight and obesity, and hyperuricemia and metabolic syndrome, as well as unhealthy habits. For all the diabetes patients the treatment should lowered BP, decreased RAS activation, prevention cholesterol rising and include glucose and lipid lowering per current guidelines [23]. In this context, our data showed that empagliflozin has positive effects in reducing SBP in the HFE group, which is in agreement with some evidences suggesting that the SGLT2i causes a remarkable reduction in BP [6, 24]. In spite of this, the precise mechanism of the BP reduction remains incompletely elucidated, with some involved mechanisms being related to natriuresis, reduced plasma volume, nonfluid weight loss, direct vascular effects [6], and RAS alternative axis [25].

Of note, hypertension is responsible for increased LV thickness as a compensatory mechanism [26]. In accordance with the current data, the HF group presented increased LV thickness and LV hypertrophy, which could be related to an increase in the LV mass and SBP, while empagliflozin treatment decreased LV thickness. Indeed, SGLT-2i is associated with improvements in regression of LV hypertrophy and cardiac remodeling and can also mitigate cardiac fibrosis remodeling [27].

Although our results showed that HF-fed mice had cardiac hypertrophy and higher SBP, the histological analysis and a specific biochemical assay showed that collagen content in the myocardium was not significantly affected in these animals, which could be due to the shorter duration of the high-fat diet in the current study. Indeed, when mice are fed a high-fat diet for longer periods, such as 24 weeks, interstitial fibrosis in the myocardium has been reported [28].

Empagliflozin reduces oxidative stress and monocyte/macrophage infiltration in the heart of metabolic syndrome rats [8]. The increase in oxidative stress promotes cardiomyocyte hypertrophy and increased expression of collagen type I and TIMP1 [11]. The latter is a glycoprotein that inhibits collagen degradation, thereby increasing its interstitial concentration. In this study, an increase in TIMP1 expression was observed in HF-fed animals, while it was reduced in treated groups. Besides, based on present data, there was an increase in antioxidant enzymes through the catalase, Sod1, and Sod2 gene expression after treatment. Few studies are investigating these enzymes in the heart of empagliflozin-treated animals, and they showed that the expressions of Sod2 [7, 29] and catalase [29] are increased while those of Nox1, Nox2 [30], and Nox4 [7] are decreased after treatment. However, this study demonstrated that empagliflozin can also downregulate the level of NOX4 in the LV of the HF-fed animals. NOX4 is the major NAD(P)H oxidase isoform in cardiomyocytes, which is associated with cardiomyopathy in the diabetes model [7]. In this way, these results indicate that empagliflozin can attenuate oxidative stress by elevating the expression of the antioxidant enzymes and reducing oxidation products in the LV of the high-fat-fed mice.

The RAS is a central component of the pathophysiological responses of cardiovascular system and has two activation routes with opposite actions, the classical pathway [14, 31] and a nonclassical axis, in which Ace2 produces angiotensin 1-7, activates the Masr, and leads to systemic arteriolar vasodilation, diuresis, reduced oxidative stress, and antiproliferative activity by increasing nitric oxide. Moreover, through the Masr, angiotensin reduces signaling pathways considered responsible for fibrogenesis and chronic inflammation [14].

Despite these facts, the role of SGLT2i on local RAS modulation in the LV is still unclear. The intrarenal RAS suppression has been demonstrated in response to SGLT2 inhibition in experimental models of T2D [32] and may contribute to the reduction in cardiovascular complications [33]. Our data suggest that empagliflozin leads to beneficial LV remodeling through decreased gene expression of the RAS classical pathway (renin, Ace1, and Atr1), in contrast to their high expression in the HF-fed mice, complying with the increased BSP and the LV hypertrophy found.

On the other hand, in the nonclassical pathway, considered as the counterregulatory axis, empagliflozin increased Atr2 and Masr gene expression in LV. Ace2 did not show any decrease in its expression, so we performed the analysis of the relationship between Ace/Ace2, which was above 1, indicating a preference for the classic pathway [34]. This ratio ensured that empagliflozin tends to act on the nonclassical pathway, leading to cardiovascular protection. It has also been reported that there is an increase in Ang II levels after the use of empagliflozin, but this increase is not harmful due to the action of this hormone via Atr2 and the concomitant increase in angiotensin 1-7 [10]. However, some studies show a decrease in RAS through a decrease in angiotensinogen and Ang II and in renin [35] excretion after dapagliflozin treatment, but the present study is the first one to ascertain the local expression of this pathway in the heart after empagliflozin use.

Regarding ER, it is responsible for the degradation of damaged proteins and organelles and plays a critical role in obesity-related cardiac injury [36] and cardiac hypertrophy [37]. Increasing Ang II due to hypertension is known to enhance the expression of ER stress-related genes such as Chop, Atf4, Bip, and Gadd45 [12]. In our study, the markers of ER stress in the LV increased in the HF group, implying ER stress in this group, which was reversed by empagliflozin. This effect has already been described after treatment with empagliflozin [38] and other SGLT2i [39].

The limitation of this study was the lack of the analysis of cardiac function, but previous studies have shown an improvement in cardiac function using SGLT2 inhibitors, and dapagliflozin attenuates the activation of the inflammasome and deterioration of left ventricular ejection fraction in BTBR mice [40]. In *ob/ob-/- mice*, empagliflozin improves coronary microvascular function and contractile performance indicated by coronary flow velocity reserve (CFVR) and fractional area change (FAC) [41]. In addition, treatment with empagliflozin has been shown to improve the left ventricular function in a model of diabetic cardiomyopathy [42] and also to reduce oxidative stress, inflammatory activity, and cardiac dysfunction in obese C57BL/6 mice [43].

In conclusion, our data showed that treatment with empagliflozin for 5 weeks decreases body mass, plasma uric acid, and systolic blood pressure in a diet-induced obesity model. By acting on the left ventricle, empagliflozin may enhance the local RAS pathway by preventing the oxidative stress and endoplasmic reticulum stress. Empagliflozin thus ameliorates the cardiac remodeling by reducing the cardiomyocyte area and left ventricle thickness, as evidenced in high-fat diet mice.

## Figures and Tables

**Table 1:** Diet compositions. Protein, mineral, and vitamin mixes of all diets are in accordance with AIN-93M. The experimental high-fat diet had added 40% of lard +10% soybean oil.

Nutrients	Diets	
	Control	High fat
Casein (≥protein 85%)	140.0	175.0
Cornstarch	620.7	347.7
Sucrose	100.0	100.0
Lard	—	238.0
Soybean oil	40.0	40.0
Fiber	50.0	50.0
Vitamin mix	10.0	10.0
Mineral mix	35.0	35.0
Antioxidant	0.008	0.060
Total (g)	1000	1000
Energy (kcal)	3802.8	5000
Carbohydrate (% energy)	76	36
Protein (% energy)	14	14
Lipids (% energy)	10	50

**Table 2:** Primers from Plin2, renin angiotensin system, endoplasmic reticulum stress, and oxidative stress.

Genes	Sequences	
*β-actin*	Forward	TGTTACCAACTGGGACGACA
*β-actin*	Reverse	GGGGTGTTGAAGGTCTCAAA
*Plin2*	Forward	AATATGCACAGTGCCAACCA
*Plin2*	Reverse	CGATGCTTCTCTTCCACTCC
*Renin*	Forward	ACCTTGCTTGTGGGATTCAC
*Renin*	Reverse	CCTGATCCGTAGTGGATGGT
*Ace*	Forward	GTGGCTGGAAGAGCAGAATC
*Ace*	Reverse	GCCTTGGCTTCATCAGTCTC
*Ace2*	Forward	CAACAGAAGCCAGACAACA
*Ace2*	Reverse	GCCTTGGCTTCATCAGTCTC
*At1r*	Forward	CCCTGGCTGACTTATGCTTT
*At1r*	Reverse	ACATAGGTGATTGCCGAAGG
*At2r*	Forward	GAAGCTCCGCAGTGTGTTTA
*At2r*	Reverse	TGGCTAGGCTGATTACATGC
*Masr*	Forward	TTCTCCACCATCAACAGCAG
*Masr*	Reverse	CCTGGGTTGCATTTCATCTT
*Atf4*	Forward	CCGAGATGAGCTTCCTGAAC
*Aft4*	Reverse	ACCCATGAGGTTTCAAGTGC
*Chop*	Forward	CTGCCTTTCACCTTGGAGAC
*Chop*	Reverse	CGTTTCCTGGGGATGAGATA
*Gadd45*	Forward	GCGAGAACGACATCAACATC
*Gadd45*	Reverse	GTTCGTCACCAGCACACAGT
*Nox4*	Forward	TCTCAGGTGTGCATGTAGCC
*Nox4*	Reverse	TTGCTGCATTCAGTTCAAGG
*Catalase*	Forward	TTGACAGAGAGCGGATTCCT
*Catalase*	Reverse	TCTGGTGATATCGTGGGTGA
*Sod1*	Forward	TGGTGGTCCATGAGAAACAA
*Sod1*	Reverse	AATCCCAATCACTCCACAGG
*Sod2*	Forward	CAGGACCCATTGCAAGGAA
*Sod2*	Reverse	GTGCTCCCACACGTCAATCC
*Timp1*	Forward	CACAGACAGCCTTCTGCAAC
*Timp1*	Reverse	CATTTCCCACAGCCTTGAAT

Abbreviations: Plin2: perilipin 2; Ace: angiotensin-converting enzyme; Ace2: angiotensin-converting enzyme 2; AT1r: angiotensin II type 1 receptor; At2r: angiotensin II type 2 receptor; Masr: Mas-receptor; Atf4: activating transcription factor 4; Chop: Caat-enhancer-binding protein homologous protein; Gadd45: growth arrest and DNA damage-inducible gene 45; Nox4: NADPH oxidase 4; Sod1: superoxide dismutase [Cu-Zn]; Sod2: superoxide dismutase [Mn]; Timp1: metallopeptidase inhibitor type 1.

**Table 3:** Biometric results, plasma and urinary analyses, and pretreatment and posttreatment.

	C	CE	HF	HFE
*Pretreatment*				
Body mass (g)	25.3 ± 0.4	—	28.9 ± 0.7^a^	—
Food intake (g)	2.5 ± 0.9	—	2.1 ± 0.4^a^	—
Water intake (mL)	4.8 ± 1.6	—	3.8 ± 0.9^a^	
OGTT (a.u.c)	863.6 ± 136.7	—	1083 ± 108^a^	—

*Posttreatment*				
Body mass (g)	24.8 ± 0.9	25.0 ± 0.9	28.8 ± 1.3^a^	26.7 ± 0.8^b^
Food intake (g)	2.6 ± 0.1	2.5 ± 0.3	2.1 ± 0.2^a^	2.6 ± 0.1^b^
Water intake (mL)	4.8 ± 0.6	5.4 ± 0.6^a^	3.7 ± 0.4^a^	4.9 ± 0.7^b^
OGTT (a.u.c)	829 ± 71.9	786.3 ± 67.6	1028 ± 25.9^a^	973.3 ± 20.1^b^

*Plasma parameters*				
HOMA-IR	12.14 ± 4.0	10.30 ± 2.3	32.2 ± 7.2^a^	17.4 ± 1.7^b^
Fasting glucose (mmol/L)	4.9 ± 0.7	4.7 ± 0.4	8.8 ± 0.7^a^	6.6 ± 0.8^b^
Plasma insulin (microU/L)	48.6 ± 15.2	55.7 ± 1.9	100.9 ± 17.8^a^	44.6 ± 3.9^b^
Plasma cholesterol (mg/dL)	117.9 ± 3.7	124.2 ± 8.0	132.8 ± 5.4^a^	107.7 ± 5.9^b^
Plasma uric acid (mg/dL)	6.1 ± 0.2	6.5 ± 0.2^a^	7.6 ± 0.5^a^	6.3 ± 0.3^b^

*Urinary parameters*				
Urinary volume (mL/24 h)	3179 ± 595.7	4530 ± 327.1^a^	1555 ± 91.9^a^	2924 ± 331.5^b^
Urinary glucose (mg/mL)	0	1200 ± 447.2^a^	0	1800 ± 447.2^b^
Urinary uric acid (mg/dL)	9974 ± 2099	13598 ± 884	5744 ± 1535^a^	8763 ± 793^b^
Left ventricle mass/tibia length (mg/cm)	0.0035 ± 0.0002	0.0037 ± 0.0001	0.0042 ± 0.0001^a^	0.0037 ± 0.002^b^

Data are presented with mean ± standard deviation, *n* = 5. C: control; CE: control+empagliflozin; HF: high fat; HFE: high fat+empagliflozin. ^a^≠C; ^b^≠HF. *p* < 0.05; one-way ANOVA and posttest Tamhane T2.

## Data Availability

The data used to support the findings of this study are available from the corresponding author upon request.
